# Multi-Tissue Omics Analyses Reveal Molecular Regulatory Networks for Puberty in Composite Beef Cattle

**DOI:** 10.1371/journal.pone.0102551

**Published:** 2014-07-21

**Authors:** Angela Cánovas, Antonio Reverter, Kasey L. DeAtley, Ryan L. Ashley, Michelle L. Colgrave, Marina R. S. Fortes, Alma Islas-Trejo, Sigrid Lehnert, Laercio Porto-Neto, Gonzalo Rincón, Gail A. Silver, Warren M. Snelling, Juan F. Medrano, Milton G. Thomas

**Affiliations:** 1 Department of Animal Science, University of California Davis, Davis, California, United States of America; 2 Commonwealth Scientific and Industrial Research Organization, Food Futures Flagship and Division of Animal, Food and Health Sciences, Queensland Bioscience Precinct, St Lucia, Queensland, Australia; 3 Department of Animal and Range Sciences, New Mexico State University, Las Cruces, New Mexico, United States of America; 4 Queensland Alliance for Agriculture and Food Innovation, Centre for Animal Science, University of Queensland, Gatton, Queensland, Australia; 5 United States Department of Agriculture, Meat Animal Research Center, Clay Center, Nebraska, United States of America; 6 Department of Animal Sciences, Colorado State University, Fort Collins, Colorado, United States of America; Leibniz-Institute for Farm Animal Biology (FBN), Germany

## Abstract

Puberty is a complex physiological event by which animals mature into an adult capable of sexual reproduction. In order to enhance our understanding of the genes and regulatory pathways and networks involved in puberty, we characterized the transcriptome of five reproductive tissues (i.e. hypothalamus, pituitary gland, ovary, uterus, and endometrium) as well as tissues known to be relevant to growth and metabolism needed to achieve puberty (i.e., *longissimus dorsi* muscle, adipose, and liver). These tissues were collected from pre- and post-pubertal Brangus heifers (3/8 Brahman; *Bos indicus* x 5/8 Angus; *Bos taurus*) derived from a population of cattle used to identify quantitative trait loci associated with fertility traits (i.e., age of first observed *corpus luteum* (ACL), first service conception (FSC), and heifer pregnancy (HPG)). In order to exploit the power of complementary omics analyses, pre- and post-puberty co-expression gene networks were constructed by combining the results from genome-wide association studies (GWAS), RNA-Seq, and bovine transcription factors. Eight tissues among pre-pubertal and post-pubertal Brangus heifers revealed 1,515 differentially expressed and 943 tissue-specific genes within the 17,832 genes confirmed by RNA-Seq analysis. The hypothalamus experienced the most notable up-regulation of genes via puberty (i.e., 204 out of 275 genes). Combining the results of GWAS and RNA-Seq, we identified 25 loci containing a single nucleotide polymorphism (SNP) associated with ACL, FSC, and (or) HPG. Seventeen of these SNP were within a gene and 13 of the genes were expressed in uterus or endometrium. Multi-tissue omics analyses revealed 2,450 co-expressed genes relative to puberty. The pre-pubertal network had 372,861 connections whereas the post-pubertal network had 328,357 connections. A sub-network from this process revealed key transcriptional regulators (i.e., *PITX2*, *FOXA1*, *DACH2*, *PROP1, SIX6, etc.*). Results from these multi-tissue omics analyses improve understanding of the number of genes and their complex interactions for puberty in cattle.

## Introduction

Puberty is the process by which animals mature into an adult capable of sexual reproduction [1]. In heifers, puberty is characterized by the dynamic and biphasic response of the hypothalamus and pituitary gland to gonadal steroids and the subsequent increase in pulsatile secretion of luteinizing hormone (LH). These endocrine patterns result in first ovulation followed by a short estrous cycle and then normal cycles thereafter [2], [3]. These events are similar in the two bovine sub-species (i.e., *Bos indicus* and *Bos taurus)*, but occurred at markedly older ages in *Bos indicus* heifers [4], [5]. However, despite a growing molecular and physiological understanding of the reproductive system, knowledge of the precise mechanisms regulating puberty in ruminants is limited, and phenotypic identification of animals that undergo puberty at an early age is costly and labor-intensive. Therefore, enhancing our understanding of the genes and regulatory pathways and networks involved in bovine puberty will provide knowledge to help improve genetic selection and reproductive management in cattle.

The first bovine genome assembly was published in 2009 [6], and since that time, the development and use of various whole genome-omics tools has accelerated investigations of various aspects of cattle genetics [7], [8]. Whole genome single nucleotide polymorphism (SNP)-chip and RNA sequencing (RNA-Seq) data from the hypothalamus were used to construct gene networks associated with puberty in cattle [9], [10], [11]. Results from these approaches allowed us to postulate that regulatory loci underlying the quantitative trait loci (QTL) associated with heifer fertility traits influence puberty. Livestock production traits are usually complex and involve multiple tissues. The construction of gene co-expression networks can therefore help identify entire groups of differentially regulated genes across the various tissues composing the reproductive-endocrine axis of mammals. This approach has been useful in studies of skeletal muscle in ruminants [12], [13], [14] and human disease [15], [16], [17].

In the present study, we characterized the transcriptome of five reproductive tissues (i.e. hypothalamus, pituitary gland, ovary, uterus, and endometrium) as well as tissues known to be relevant to growth and metabolism and needed for cattle to achieve puberty (i.e., *longissimus dorsi* muscle, adipose, and liver). These tissues were collected from pre (PRE)- and post (POST)-pubertal Brangus heifers (3/8 Brahman x 5/8 Angus) that were progeny of a pedigreed-population of cattle used to identify QTL associated with fertility [11], [18], [19]. The fertility traits were age of first observed *corpus luteum* (ACL), first service conception (FSC), and heifer pregnancy (HPG). The first trait is quantitative and the other two are binary. A heifer that records success for these traits is considered to have experienced early puberty. This age requirement is a challenge for *Bos indicus*-influenced heifers [2], [4], [11], [19]. The QTL associated with these traits were determined with genome-wide association studies (GWAS). In order to exploit the power of complementary omics analyses, PRE and POST puberty co-expression gene networks were constructed by combining the results from GWAS and RNA-Seq (i.e., differential expression (DE) and tissue specific expression (TS)). The knowledge of transcription factors (TF) and network theory framework also contributed new insights into the regulatory genes (i.e., hubs) within these networks.

## Results and Discussion

### RNA-Seq data and normalization

**Figure 1:**
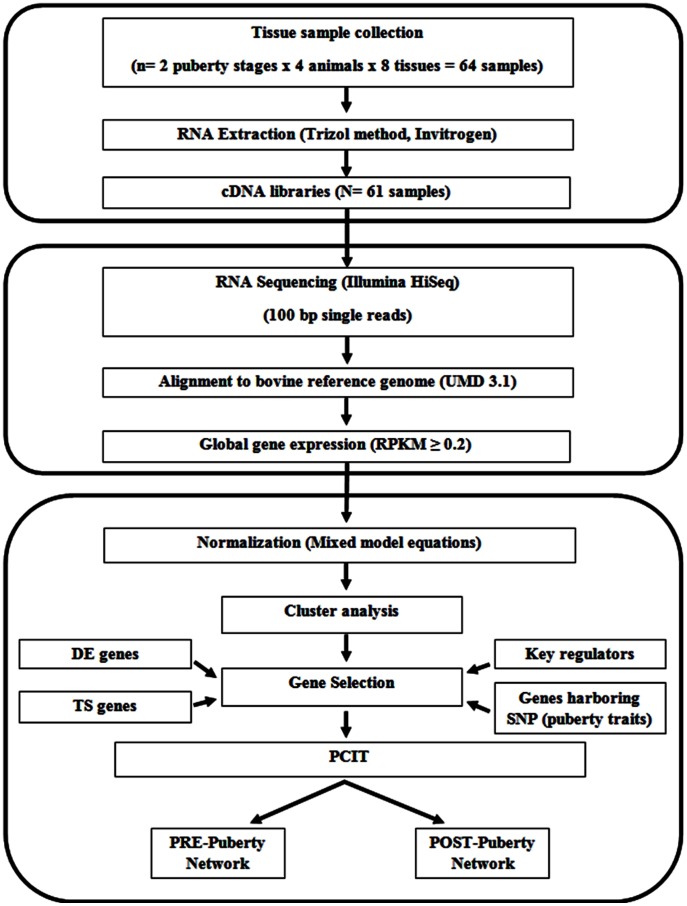
Flowchart of the analytical steps from tissue collection to RNA-Seq to normalization and network construction. DE (differentially expressed genes); RPKM (reads per kilobase of exon per million reads); TS (tissue specific); PCIT (partial correlation and information theory). Note: We failed to process three tissue samples, so 61 tissues were used for RNA-Seq analyses.

Sixty-one samples from PRE (n = 4) and POST (n = 4) Brangus beef heifers were analyzed with RNA-Seq (Figure 1). Samples were harvested from animals handled and managed according to the Institutional Care and Use Committee of New Mexico State University (approval number 2010-013). An average of 30 million sequence reads was obtained from each sample. These sequence results were assembled and mapped to the annotated 27,368 genes in the bovine genome assembly UMD3.1.74.

The relatively high number of reads from each cDNA library used in this study provided the sensitivity needed to detect lowly expressed genes. This statement is based on the conclusions of the studies described in Rapaport et al [20]. Specifically, differential expression of genes as measured with RNA-Seq provides enhanced sensitivity for detection of lowly expressed genes. Also, our prior experience analyzing RNA-Seq data provided strong evidence that our read-depth and number of libraries per tissue were adequate for this type of study [21], [22], [23].

**Figure 2:**
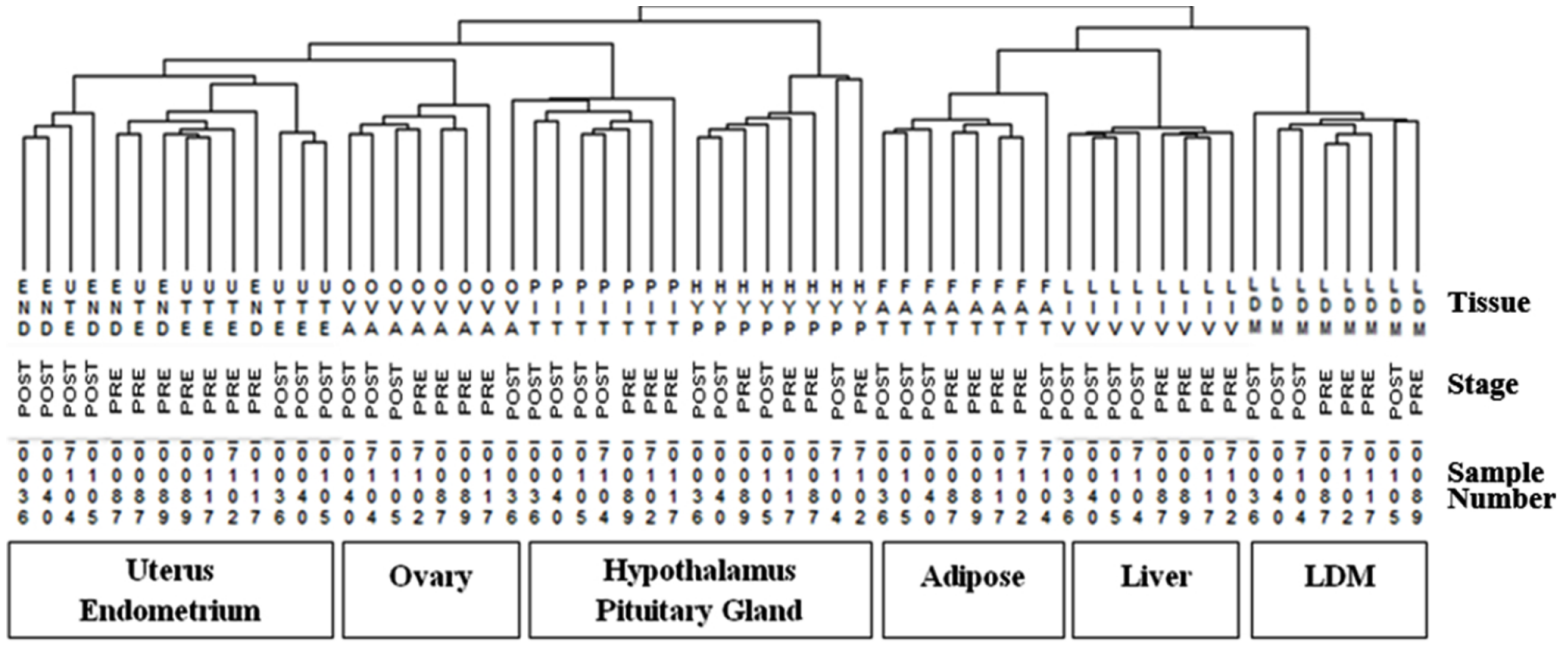
Hierarchical clusters from RNA-Seq data of 17,832 genes across 61 tissues. Samples clustered first by tissue of origin and then by stage, PRE or POST-puberty. Abbreviations of samples include: HYP (hypothalamus); PIT (pituitary gland); UTE (uterus); END (endometrium); OVA (ovary); FAT (adipose); LIV (liver); LDM (*longissimus dorsi* muscle).

In the current study, 70 to 80% of the sequences were categorized as mapped reads to the bovine reference assembly. Analyses of the reads per kilobase of exon per million reads (RPKM values) of sequence [24] were used to establish the total number of genes expressed in the transcriptome of the hypothalamus, pituitary gland, liver, *longissimus dorsi* muscle, adipose, uterine horn, endometrium, and ovary. Approximately 65% of the bovine transcriptome was represented in at least one tissue and physiological state (17,832 genes out of a total of 27,368 annotated *Bos taurus* genes). Hierarchical cluster analysis validated the optimality of RNA-Seq data normalization procedures. Figure 2 shows that RNA-Seq data clustered first according to tissue, and then according to developmental stage (PRE and POST). The number of unique reads and RPKM of each gene within each of the 61 samples are publically available at the Gene Expression Omnibus (GEO; http://www.ncbi.nlm.nih.gov/geo/; accession number GSE55435). Table S1 list the 2,450 genes that will be discussed in the following sections. Specifically, this table provides average gene expression level for DE, TS, TF, and (or) containing a SNP detected with GWAS in PRE and POST heifers.

### Differentially expressed genes among PRE and POST puberty heifers

The statistical significance of differential gene expression was ascertained via mixtures of distributions. The two-component mixture model was applied to the vector of differential expression measures in all genes simultaneously. However, for each gene, the p-values correspond to the posterior probability of belonging to each component in the mixture, the component with non-differentially expressed genes (clustered around zero and with small variance) and the component with differentially expressed genes (also clustered around zero, but with large variance allowing to capture extreme values).

**Figure 3:**
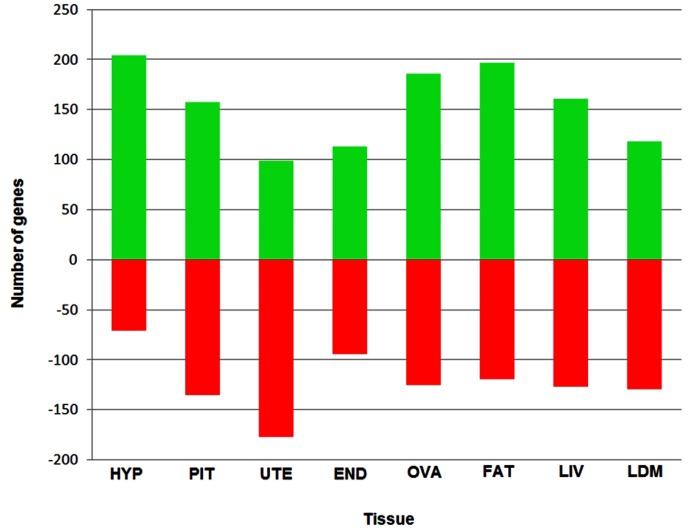
Genes down- and up-regulated between PRE and POST puberty heifers across eight tissues. Number of genes down- (red) and up-regulated (green) from the 2,212 differentially expressed genes between PRE and POST puberty heifers (corresponding to 1,515 unique genes) detected with RNA-Seq analysis. Abbreviations include: HYP (hypothalamus); PIT (pituitary gland); UTE (uterus); END (endometrium); OVA (ovary); FAT (adipose); LIV (liver); LDM (*longissimus dorsi* muscle).

Resulting from this approach, a total of 2,212 transcripts corresponding to 1,515 annotated genes were found to be DE (*P*<0.001) among PRE and POST heifers in at least one of the eight tissues. Figure 3 shows the numbers of genes differentially up- and down-regulated for each of the eight tissues among PRE and POST heifers. The highest proportion of up-regulated genes was observed in the hypothalamus as 204 of the 275 DE genes were up-regulated in heifers reaching puberty. In adipose tissue, 197 genes were up-regulated and 119 genes were down-regulated. This tissue therefore had the largest overall number of DE genes (n = 316) between the PRE and POST states. These results were expected as the endocrine response of the hypothalamus to signals from adipose tissue have been a long-term focus of study for cattle puberty [3], [25], [26].

Arginine vasopressin (*AVP*) and oxytocin (*OXT*) were the most significantly DE genes in hypothalamus. The *AVP* gene was also DE between PRE and POST heifers in adipose, liver, ovary, and uterus (up-regulated in all) while *OXT* showed up-regulation in adipose, hypothalamus and ovary and down-regulation in *longissimus dorsi* muscle. Arginine vasopressin and oxytocin are posterior pituitary peptide hormones, which are synthesized in the supraoptic and paraventricular nuclei of the hypothalamus. These hormones are involved in many physiological events such as lactation, renal and cardiovascular functions, as well as cognition, tolerance, adaptation, and complex sexual and maternal behavior [27], [28]. However, this is an interesting observation as adipose tissue is generally considered an endocrine target of *OXT*. The vasoactive intestinal peptide (*VIP*) gene was found DE in hypothalamus and pituitary gland (up regulated). This gene was also associated with ovarian development [29], [30].

The pituitary gland had 292 DE genes (i.e. 135 down- and 157 up-regulated, respectively). The largest DE was observed for the genes of ribosomal protein L39 gene (*RPL39*) essential for cell growth development and Vitamin K-dependent protein Z (*PROZ*) known to be involved in the venous thrombosis and pregnancy in several human populations [31], [32]. The *RPL39* gene was initially associated with reduced body size and diminished fertility [32], [33] and more recently with testicular function [34]. This gene was also DE in hypothalamus and *longissimus dorsi* muscle (up-regulated at puberty). The relaxin gene, specifically *RLN3,* was also observed in the list of the most down-regulated genes in the pituitary gland. This gene is expressed in several reproductive tissues as it has various roles in the female such as softening of the cervix, elongation of the pubic symphysis, inhibition of uterine contractions at parturition, as well as unique roles in enhancing sperm motility. Regarding the pituitary gland, relaxin has a role in regulating secretion of oxytocin and vasopressin [35], [36].

In samples from the uterine horn and endometrium, 207 and 276 DE genes respectively were detected among PRE and POST heifers. The teratocarcinoma-derived growth factor 1 (*TDGF1*) and proenkephalin (*PENK*) genes were the most DE genes observed and both have been shown to play an essential role in embryonic development, the estrous cycle, and early pregnancy, which coincide with the strong body of evidence for the role of *PENK* in neural tissues [37], [38]. *TDGF1* and *PENK* were up-regulated in the POST heifers in five out of eight tissues analyzed (uterus, endometrium, hypothalamus, pituitary gland and liver). The *PENK* gene maps to a region on bovine chromosome 14, which has been shown to be associated with fertility traits in this population of Brangus cattle as well as Brahman cattle of Australia [19], [39], [40].

The SIX homeobox 6 (*SIX6*) and PROP paired-like homeobox 1 (*PROP1*) genes also appeared as some of the most significantly up-regulated genes in the endometrium. The *SIX6* gene was also up-regulated in the hypothalamus and liver, consistent with its postulated role as an important regulator of gonadotropin-releasing hormone (GnRH) [41]. This is an interesting result as GnRH is now known for its role in a plethora of tissues [42]. The *PROP1* gene has roles in pituitary development and hormone expression such as luteinizing hormone, follicle-stimulating hormone, growth hormone, prolactin, and thyroid-stimulating hormone [43], [44], [45].

The number of DE genes detected in the liver and ovary were 288 and 311, respectively. In the ovary, 186 genes were up-regulated and 125 were down-regulated. The liver had a higher proportion of genes up-regulated (n = 161) versus down-regulated (n = 127) with the neuregulin 3 (*NRG3*) gene as one of the most up-regulated genes in POST heifers. This gene encodes ligands for the transmembrane tyrosine kinase receptors members of the epidermal growth factor (EGF) receptor family. It also promotes mammary differentiation during embryogenesis [46]. Differential expression of *DHRS9* (dehydrogenase/reductase (SDR family) member 9) gene was observed among PRE and POST heifers. Dysregulation of several genes involved in lipid metabolism and Wnt signaling involve *DHRS9* and it has been associated with abnormal ovary development and function [47].

Enrichment analyses of GO terms were performed using the 1,515 DE genes and the genes expressed in at least one tissue and physiological state (17,832 genes) as a background list. As expected of PRE versus POST heifers and the reproductive axis, GO terms related with hormone activity, receptor activity and neuropeptide receptor binding were abundant (Table 1). The molecular function of GO terms included hormone activity, G-protein coupled receptor activity, and serine-type endopeptidase activity (*P*<4.61E-13). Additionally, a variety of molecular functions related to cell signaling activity, receptor binding and receptor activity were also present in the list of most significant GO terms including the highest proportion of DE genes (from 121 to 148 DE genes across tissues and physiological states; Table 1).

**Table 1:** Top ten molecular function gene ontology (GO) terms significantly enriched among the 1,515 differentially expressed unique genes across the tissues.

GO term	Description	P-value	FDR q-value	DE Genes
GO:0005179	Hormone activity	2.4E-19	9.23E-16	39
GO:0004930	G-protein coupled receptor activity	1.12E-13	2.15E-10	74
GO:0004252	serine-type endopeptidase activity	4.61E-13	5.91E-10	37
GO:0004888	Transmembrane signaling receptor activity	6.65E-13	6.4E-10	112
GO:0008236	Serine-type peptidase activity	7.42E-13	5.71E-10	40
GO:0017171	Serine hydrolase activity	1.25E-12	8.03E-10	40
GO:0038023	Signaling receptor activity	2.5E-12	1.37E-09	121
GO:0005102	Receptor binding	7.74E-12	3.73E-09	148
GO:0004872	Receptor activity	2.14E-11	9.15E-09	138
GO:0030594	Neurotransmitter receptor activity	6.95E-11	2.67E-08	26

Serine-type endopeptidase activity, serine-type peptidase activity, and serine hydrolase activity were also among the most enriched GO terms (Table 1). The serine protease family of genes includes plasminogen activator inhibitor (*PAI2*) secreted by the placenta and used as maternal biomarkers and early fetal size in humans. Genes related to fat metabolism and hormonal processes (*ADIPOQ*), growth (*GH1 and VGF*), regulation of body weight (*LEP*), and formation of the mammary gland (*PTHLH*) were also observed. Several of these genes have been used in both physiology and genotype to phenotype association studies related to growth and reproduction in the population of Brangus cattle from which the heifers of the current study were selected [48], [49], [50].

### Tissue specific genes

Nine hundred and forty-three genes were found to have TS expression (*P*<0.001) in at least one of the eight tissues. Endometrium (n = 142), uterus (n = 132), pituitary gland (n = 122), ovary (n = 166) and *longissimus dorsi* muscle (n = 170) expressed more than 100 TS genes while the number of TS genes in hypothalamus, liver and fat ranged from 85 to 96. The ovary and endometrium expressed the highest number of TS genes and many of these genes were also found to be DE between PRE and POST heifers. An example of such a gene was the insulin-like peptide 3 (*INSL3)* gene, which is transcribed in gonadal tissues [51], [52].

Myosin heavy chain 1 (*MYH1*), fatty acid binding protein 4 (*FABP4*), and adiponectin (*ADIPOQ*) were the most significant TS genes expressed in *longissimus dorsi* muscle and subcutaneous fat. Myosin is involved in contraction, while *FABP4* encodes the fatty acid binding protein found in adipocytes. The functions of FABP proteins include fatty acid uptake, transport, and metabolism [53], [54]. Also, *FABP4* has been associated with marbling and carcass weight in cattle [55]. Changes in the gene expression of *ADIPOQ* and its receptors were associated with ovarian follicular recruitment and luteal function in Holstein cows [56]. Cumulatively, these results provide gene-specific information to support the rationale for long-term study of the relationships of adiposity with fertility in Brangus cattle [57], [48], [58].

A GO enrichment analysis was performed using the 943 TS genes. Sequence-specific DNA binding and nucleic acid binding transcription factor activity was amongst the most significantly enriched GO terms (Table 2). G-protein coupled receptor activity, structural constituents of muscle, structural molecule activity, peptidase inhibitor activity, and neurotransmitter receptor activity should also be noted. Hormone activity was an expected GO term to be observed in this analysis as half the tissues from which RNA was extracted have known endocrine activities. Of the 23 genes composed in this list, notable gene observations included *PTHLH*, *UTMP*, and *FSH*. The first two genes in this list have roles in development of females for organs such as the mammary and the reproductive tract-uterus [59], [60], [61] and expression was observed in uterus and endometrium. Follicle stimulating hormone (FSH) is the well-characterized dimeric-glycoprotein synthesized and secreted by the anterior pituitary gland to stimulate ovarian follicular growth and steroidogenesis [62], [63].

**Table 2:** Top ten molecular function gene ontology (GO) terms significantly enriched among the 943 tissue-specific (TS) unique genes (across all tissues).

GO term	Description	P-value	FDR q-value	TS Genes
GO:0043565	Sequence-specific DNA binding	1.8E-17	6.92E-14	86
GO:0003700	Sequence-specific DNA binding transcription factor activity	1.12E-11	2.15E-08	97
GO:0001071	Nucleic acid binding transcription factor activity	1.28E-11	1.64E-08	97
GO:0004930	G-protein coupled receptor activity	5.03E-11	4.84E-08	53
GO:0008307	Structural constituent of muscle	2.22E-10	1.71E-07	16
GO:0005179	Hormone activity	4.08E-10	2.62E-07	23
GO:0000976	Transcription regulatory region sequence-specific DNA binding	7.3E-07	4.01E-04	24
GO:0005198	Structural molecule activity	7.31E-07	3.51E-04	57
GO:0030414	Peptidase inhibitor activity	9.84E-07	4.21E-04	22
GO:0030594	Neurotransmitter receptor activity	2.1E-06	8.06E-04	16

### Identification of key gene regulators

**Figure 4:**
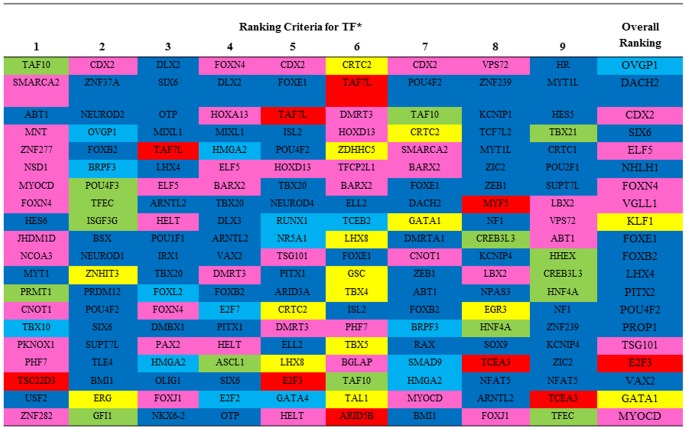
Relevant Transcription Factors (TF): Top 20 (for nominal 5% significance) transcription factors from the 364 included in the network according to nine criteria^*^. Colors represent tissue of maximum expression: hypothalamus and (or) pituitary gland (dark blue), ovary (light blue), uterus and (or) Endometrium (pink), liver (green), *longissimus dorsi* muscle (red), adipose (yellow).^*^Ranking criteria are: 1) RIF1; 2) RIF2; 3) Overall differential expression; 4) Maximum differential expression (DE); 5) Connections in PRE-puberty network; 6) Connections in POST-puberty network; 7) Fold change in connections between PRE- and POST-puberty networks; 8) Network expansion ability in PRE-puberty; and 9) Network expansion ability in POST-puberty.

Regulatory impact factor (RIF) and transcription factor binding sites (TFBS) approaches were used to identify regulators with the highest evidence contributing to DE and (or) TS gene specificity in PRE and POST heifers. Using the RIF metrics, which exploits the concept of differential co-expression (see Materials and Methods), 1,329 regulators were contrasted against a unique list of genes that were either DE or TS, identifying 221 TF. A search for motifs corresponding to TFBS of known TF identified 143 TF. Combining the results from RIF (221 TF) and TFBS (143 TF) approaches, 364 TF were identified contributing to DE and (or) TS among PRE and POST heifers. Figure 4 shows the top 20 TF from these efforts.

The highest proportion of up-regulated genes between PRE and POST heifers was observed in the hypothalamus (Figure 3). There have been several attempts to construct gene networks to understand the role of the hypothalamus in on-set of puberty in several model animals [64], [65], [66]. In the cattle of the current study, 11 of the 20 most highly ranked TF were highly expressed in hypothalamus and (or) pituitary gland (Figure 4). Among them, *OVGP1* (oviductal glycoprotein 1), *DACH2* (dachshund homolog 2), *CDX2* (caudal type homeobox 2) and *SIX6* (SIX homeobox 6) were the highest ranked TF. These genes are also involved in follicular and embryonic development (*OVGP1*), premature ovarian failure (*DACH2*), early embryonic development of the intestinal tract (*CDX2*), and regulation of gonadotropin-releasing hormone expression (*SIX6*) [41], [67]. Our results show *SIX6* as one of the most relevant TF as its data values were maximized in hypothalamus and (or) pituitary gland. Other noted TF included the forkhead family of transcription factors such as *FOXN4*, *FOXE1* and *FOXB2* as they appeared in the middle of the overall ranking (Figure 4). These TF are involved in a variety of biological processes such as body development and metabolism [68], [69].

**Figure 5:**
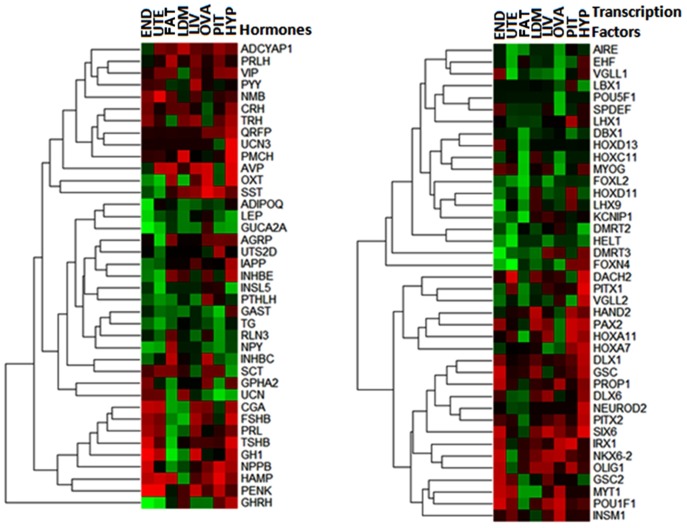
Heat maps of the differential expression in PRE versus POST pubertal heifers across tissues. Left panel: differential expression (DE) of 39 hormones. Right panel: differential expression of 40 tissue-specific (TS) transcription factors. Abbreviations of tissues: HYP (hypothalamus); PIT (pituitary gland); UTE (uterus); END (endometrium); OVA (ovary); FAT (adipose); LIV (liver); LDM (*longissimus dorsi* muscle). The spectrum goes from bright green (down-regulation) to bright red (up-regulation).

Reproduction involves an endocrine axis and many genes are regulated by TF; therefore, heat maps of hierarchical cluster analyses involving hormones and TF among PRE and POST heifers were constructed (Figure 5). Note that autoimmune regulator (*AIRE*), dachshund homolog 2 (*DACH2*) and forkhead box L2 (*FOXL2*) were observed as differentially expressed TF among PRE and POST heifers. These TF are involved in ovarian development and function as well as oocyte gene expression [70], [71], [72]. Both *AIRE* and *FOXL2* were down-regulated at puberty in the eight tissues while *DACH2* was up-regulated in hypothalamus via puberty.

The homeobox genes encode a highly conserved family of transcription factors such as *HOXD13*, *HOXC11*, *HOXD11*, *HOXA11* and *HOXA7* (Figure 5). These TF have an important role in morphogenesis in all multicellular organisms and have been associated with severe limb and genital abnormalities (*HOXD11* and *HOXD13*), early intestinal development (*HOXC11*), and regulation of uterine development and fertility (*HOXA11*) [72], [73], [74]. Most of the HOX family genes were down regulated at puberty across eight tissues with the exception of *HOXA11* and *HOXA7*, which were up-regulated in the hypothalamus and pituitary gland post puberty.

### Identification of genes harboring SNP

We used GWAS results from three recent studies of heifer fertility traits (i.e., ACL, FCS, and HPG) to yield a list of genes harboring SNP with significant associations to the timing of puberty [11], [39]. This list was compared with the 17,832 genes observed in at least one tissue among the two physiological states evaluated with RNA-Seq. This effort identified 235 genes.

Table 3 is a list of genes harboring SNP associated with heifer fertility traits observed with a GWAS and detected as either DE or TS genes with RNA-Seq analyses. Combining these results allowed the identification of 25 QTL associated with ACL (11 QTL), FSC (4 QTL) and HPG (10 QTL). Among them, 19 out of the 25 QTL were detected as DE genes. Of the eight SNP that were within 10 Mb of an annotated gene, 5 were found to be upstream in the promoter enhancer region and 3 were found to be downstream in the 3′ untranslated region. Note that the *PENK* (proenkephalin) gene was DE among PRE and POST heifers and expressed in the pituitary gland. In our DE analyses, this gene was also detected in uterus, endometrium, hypothalamus, and liver (i.e., five out of eight tissues analyzed). The *PENK* gene is part of the opioid system, which influences the axes composed of the hypothalamus, pituitary gland, and both the gonad and adrenal gland [35], [37], [38].

**Table 3:** Genes harboring SNP associated with heifer fertility traits and with expression found to be either differentially expressed (DE) or tissue specific (TS) in RNA-Seq analysis of tissues from pre- and post-pubertal heifers.

RNA-Seq Analysis					SNP Association Analysis					
Gene	DE	TS	Tissue	Fold Change	SNP	Chr.	Position (bp)	Distance (bp)	Trait	P-value
DPPA4	1	0	HYP	−4.82	rs41571491	1	54,523,895	5,517^d^	HPG	0.00554
TP63	1	1	ADIPOSE	5.06	rs109034747	1	78,088,393	0	FSC	0.00745
INHA	0	1	OVA	−1.26	rs136318374	2	108,231,291	1,460^d^	ACL	0.00504
IL22RA1	1	0	END	−16.78	rs29019426	2	129,348,719	2,978^u^	FSC	0.00205
RHEBL1	1	0	PIT	−3.97	rs110770702	5	30,912,029	7,398^u^	HPG	0.00372
LYSB	0	1	END	−2.02	rs110181782	5	44,556,197	0	ACL	0.00517
ADH6	1	0	END	25.0	rs41612964	6	26,799,276	0	HPG	0.00615
MEPE	1	1	PIT	4.71	rs41650773	6	38,286,952	0	HPG	0.00590
TECRL	0	1	LDM	−1.22	rs109759346	6	81,577,343	0	ACL	0.00746
LOC777593	1	0	END	15.96	rs109621209	7	61,683,533	0	HPG	0.00549
MGC157266	1	0	END	−3.76	rs136828274	8	62,927,382	8,411^u^	ACL	0.00715
C10H11ORF46	1	1	HYP	−4.16	rs109705635	10	4,882,326	0	ACL	0.00292
NRXN3	1	0	END	−3.92	rs43651752	10	91,751,584	0	HPG	0.00283
TSHR	1	0	END	4.55	ss117964119	10	93,528,030	0	HPG	0.00369
NEBL	1	0	END	5.63	ss61500113	13	22,476,667	0	FSC	0.00157
MOS	0	1	OVA	−2.05	rs110243083	14	24,973,324	2,465^u^	ACL	0.00017
PENK	1	0	PIT	4.54	rs134428213	14	25,218,861	0	ACL	0.00061
ELF5	1	0	UTE	−17.19	rs133233558	15	65,840,403	0	ACL	0.00879
POU4F2	0	1	END	−2.45	rs41838669	17	11,828,433	1,818^u^	HPG	0.00707
FAM19A4	1	0	LIV	−4.46	rs136177962	22	32,820,574	0	ACL	0.00712
ITIH1	1	0	OVA	3.48	rs110723544	22	48,663,639	0	HPG	0.00567
CPNE5	1	0	END	−4.95	rs109264326	23	10,690,330	0	ACL	0.00743
MMD2	1	0	UTE	19.89	rs135797510	25	39,622,260	0	ACL	0.00493
DKK1	1	0	UTE	11.65	rs29024937	26	6,860,529	4,762^d^	FSC	0.00817
SYCE1	0	1	LIV	1.02	rs41629412	26	50,517,416	0	HPG	0.00743

u SNP upstream from gene, ^d^SNP downstream from gene. Abbreviations include: DE (differentially expressed); TS (tissue specific); HPG (heifer pregnancy); FSC (First service conception); ACL (Age at puberty as measured by the presence of the first *corpus luteum*); HYP (hypothalamus); PIT (pituitary gland); UTE (uterus); END (endometrium); OVA (ovary); FAT (adipose); LIV (liver); LDM (*longissimus dorsi* muscle).

The chromosomal locus containing *PENK* is also associated with ACL [10], [40]. Single nucleotide polymorphisms located in close proximity to the *MOS* (v-mos Moloney murine sarcoma viral oncogene homolog) gene were associated with ACL. This gene is involved in oocyte maturation [75] and was DE among the PRE and POST heifers in ovarian tissue. Two other transcription factors, *ELF5* and *POU4F2* were identified with high levels of expression in endometrium, hypothalamus, and pituitary gland (Figure 4). These genes were also found to be associated with ACL and HPG (Table 3). This is an interesting result as these genes are considered oncogenes that may interact with the estrogen receptor [76], [77] and therefore may influence the biphasic response of the hypothalamus to estrogen through the pubertal transitioning process [1], [3], [26]. Even though the hypothalamus was such a reactive tissue to puberty, it should be noted that 13 of the loci detected by combining results of GWAS and RNA-Seq revealed the uterus and endometrium as the primary tissues expressing the candidate gene. Furthermore, three of the SNP were found to be expressed within the ovary (Table 3). In particularly, *INHA* (inhibin alpha) which has various polymorphisms associated with Holstein cow response to superovulation and is well known for its role in regulating follicle stimulating hormone secretion [78]. This gene was also reported in the GWAS-gene identification efforts of Fortes et al. [11].

### PRE and POST puberty gene networks

**Figure 6:**
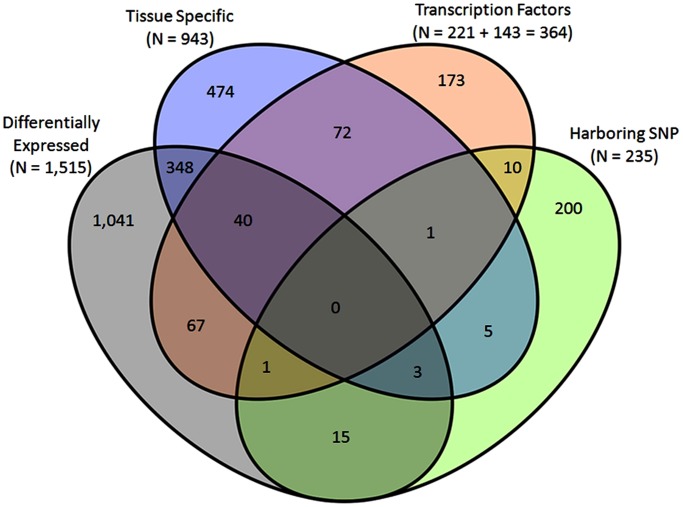
Venn diagram of 2,450 genes used to construct gene networks. Strategies used to construct gene networks involved genes that were (1) Differentially expressed (DE) between pre- and post-puberty, (2) Tissue specific (TS), (3) Transcription factors (TF), (4) Genes harboring SNP observed with GWAS and associated with indicator traits of puberty (age at puberty as measured by the presence of the first *corpus luteum* (ACL), first service conception (FSC), and heifer pregnancy (HPG)).

Gene networks offer a platform to systematically quantify and visualize perturbations of gene interactions encompassing pathways that infer phenotypes [16], [17]. Complex traits, which are typical of livestock production, usually involve multiple tissues; therefore, co-expression networks allow evaluation in genes across physiological systems and states (PRE vs POST). This strategy has proven effective in our initial studies involving skeletal muscle [12], [14], [79]. Since puberty is a complex event involving multiple tissue and physiological systems (reproduction and metabolism), gene networks were constructed using results from GWAS, RNA-Seq of eight tissues, and the knowledge of transcriptional regulators in a network theory framework. Hence “informative” genes to reverse engineer the networks were selected from four sources of information: 1) DE genes (n = 1,515) between PRE vs POST; 2) TS genes (n = 943) of eight tissues; 3) key regulators (n = 364) identified with RIF and TFBS analyses; and 4) genes harboring SNPs (n = 235) associated with ACL, FSC, and (or) HPG observed with GWAS. Thus, 2,450 unique genes were used to construct PRE and POST gene co-expression networks involving eight tissues. Figure 6 is a Venn diagram of the genes detected among these four sources of information/categories. Note that there are numerous genes that fit two of these categories, but no one gene was observed in all four categories.

**Figure 7:**
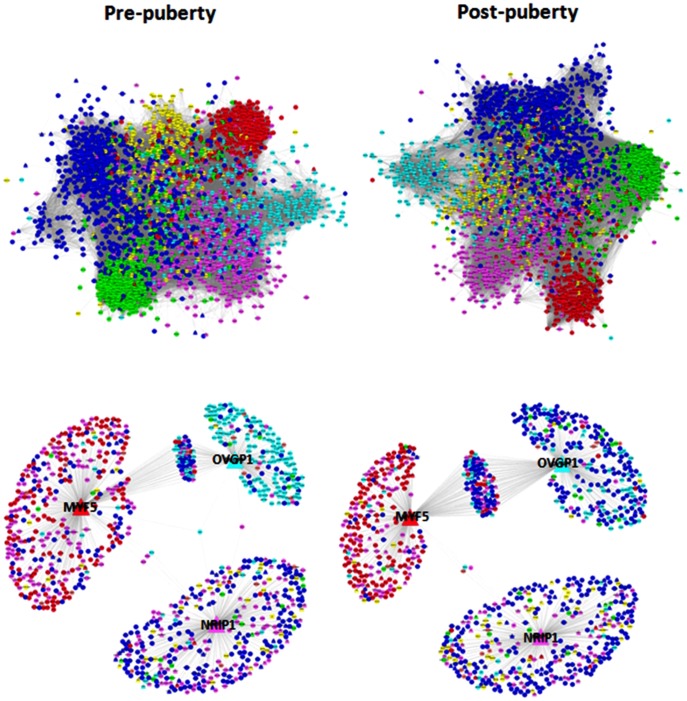
Gene co-expression networks constructed by combining results from RNA-Seq and GWAS with the knowledge of transcription regulators for pre-PRE (left panels) and post-pubertal (right panel) heifers. Upper panels correspond to the visualization of the entire network comprising 2,450 nodes (or genes) while the lower panels correspond to the expansion of trio of TF comprised of *OVGP1*, *NRIP1* and *MYF5*, defined as the best trio in terms of their ability to expand the majority of the topology of the entire networks. Colors represent tissue of maximum expression: hypothalamus and/or pituitary gland (dark blue), ovary (light blue), uterus and/or endometrium (pink), liver (green), *longissimus dorsi* muscle (red), adipose (yellow).

Pre- and post-puberty co-expression gene networks are presented in Figure 7. The PRE network had 372,861 connections for the 2,450 genes, whereas the POST network had 328,357 connections. Note the differences in the patterns among the tissues comprising the two networks. Specifically, the liver and *longissimus dorsi* muscle had an abundance of connections within the networks (i.e., ∼40% and ∼25%, respectively), while adipose and uterus had very low percentage of connections (∼2%; Table 4). This table also provides information about the number of connections that disappeared or emerged among the PRE and POST states. More connections emerged rather than disappeared in the hypothalamus, pituitary gland, endometrium, and *longissimus dorsi* muscle. The hypothalamus appeared to have gained the largest number of connections via the on-set of puberty (i.e., 30.4%), whereas the liver experienced the largest disappearance of connections. The *longissimus dorsi* muscle appeared to have a similar rate of disappearance and emergence connections among the two physiological states. This large gain in gene numbers by the hypothalamus was expected based on knowledge of the role of this tissue in regulating puberty [26], [65], [66].

**Table 4:** Tissue distribution and connectivity structure of the 2,450 genes used to build the PRE- and POST-Puberty networks.

Tissue	Tissue of Maximum Expression		% Connections in Network		% Connections that at Puberty	
	N	%	PRE-Puberty	POST-Puberty	Disappear	Emerge
Endometrium	324	13.2	6.3	4.5	8.9	13.9
Fat	219	8.9	2.5	1.8	2.4	0.1
Hypothalamus	510	20.8	16.9	21.0	15.2	30.4
Liver	358	14.6	38.2	36.3	42.5	22.1
*Longissimus dorsi* muscle	273	11.1	23.7	23.1	15.1	15.8
Ovary	301	12.3	3.4	2.6	8.9	3.9
Pituitary Gland	309	12.6	6.5	9.4	3.4	11.1
Uterus	156	6.4	2.4	1.2	3.6	2.6

**Figure 8:**
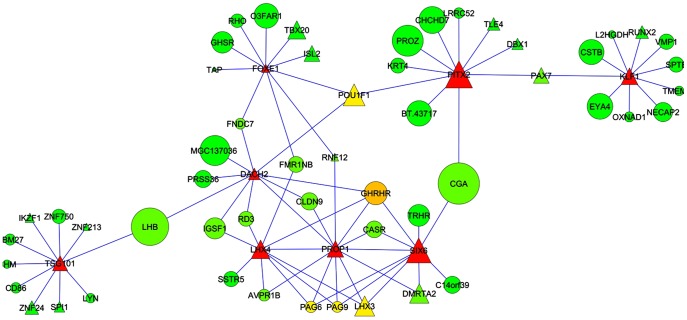
Sub-network created with highly co-expressed genes with significant hypothalamic and pituitary gland differential expression. Triangles represent transcription factors. Color indicate connectivity degree from green (low connectivity) to yellow (medium connectivity) to red (high connectivity). Size indicates the relative amount of expression in the post-puberty sample.

In order to identify potential regulators of the predicted networks, we focused on the 364 TF contained in the network. We applied an information lossless approach that explored all the TF trios (having 7,971,964 possible trios) and identified the TF trio that, through their connected genes, spanned most of the network topology with minimum redundancy. The expansion of the best trio of TF for PRE and POST gene co-expression networks is presented in the lower panels of Figure 7. The transcription factors *OVGP1*, *NRIP1* and *MYF5*, were defined as the best trio of TF in terms of their ability to expand the majority of the topology of the entire networks. Different patterns were observed in the expansion of the best trio of TF among PRE and POST co-expression networks. In brief, results revealed that the hypothalamus, pituitary gland, skeletal muscle and ovary were the tissues with the maximum expression in PRE networks, while the hypothalamus, pituitary gland and skeletal muscle were the tissues with the maximum expression in the POST network (Figure 7). In addition, a sub-network with highly co-expressed genes and significantly DE genes in the hypothalamus and pituitary gland was constructed (Figure 8). This sub-network suggested a different degree of connectivity among highly co-expressed genes and TF. The highest degree of connectivity was observed in TF such as *PITX2*, *FOXA1*, *TSG1D1*, *DACH2*, *LHX4*, *PROP1* and *SIX6*. These types of results parallel the reports of Ojeda and co-workers investigating TF and oncogenic factors influencing puberty in model organisms [64], [65], [66] and improved our initial bovine gene networks constructed with only GWAS and hypothalamus RNA-seq results [11]. Also, six genes captured in the cattle network are concordant with the human network that reported 30 loci for age at menarche (Table S2) [80].

### Concluding remarks

Analysis of RNA-Seq of eight tissues among PRE and POST Brangus heifers revealed 1,515 DE and 943 TS genes within the 17,832 genes with an RPKM >0.2. The hypothalamus experienced the most notable up-regulation of genes upon puberty among the eight tissues involved in reproduction (i.e., hypothalamus, pituitary gland, uterus-endometrium, and ovary) and in growth and metabolism (i.e., liver, muscle, adipose). As this study was conducted with heifers from a cattle breeding population, it allowed the combination of RNA-Seq and GWAS results with knowledge of transcription factors for construction of gene co-expression networks.

Combining results of GWAS with RNA-Seq identified 25 SNP-loci inferring QTL associated with fertility traits indicative of early puberty in *Bos indicus*-influenced heifers (i.e., ACL, FSC, HPG). Seventeen of these SNP were within genes and 13 of these genes were expressed in uterus or endometrium. Furthermore, the various omics analyses revealed 2,450 unique genes relevant to puberty and a sub-network of key transcription factors (i.e., *PITX2*, *FOXA1*, *TSG1D1*, *DACH2*, *LHX4*, *PROP1* and *SIX6*) regulating puberty. Results from these multi-tissue omics analyses improve understanding of the number of genes and their complex interactions for puberty in cattle. These results also help discovering genes that contain biologically relevant SNP-genotypes that can be used in genetic improvement processes of *Bos indicus*-influenced composite cattle.

## Materials and Methods

### Animals, management, and puberty

Heifers were handled and managed as per approval of the Institutional Animal Care and Use Committee of New Mexico State University (protocol #2010-013). Eight Brangus (3/8 Brahman x 5/8 Angus) heifers representing the pedigree diversity observed in GWAS of fertility traits were selected for this study from the New Mexico State University Brangus breeding program [11], [18], [19], [58]. Averages for fertility trait measures indicative of early puberty in this study were: ACL (651 days), FSC (53.3%), and HPG (78.0%)[10], [11], [19], [58].

Pubertal states were defined using circulating concentrations of progesterone measured with a radioimmunoassay (i.e., five assays with intra- and interassay CV of 4.8 and 4.3%) [81]. Bloods samples were collected twice per week (i.e., Tuesday and Friday) during the post-weaning period and four heifers were determined to be pre-pubertal (i.e., PRE; progesterone values less than 1 ng/mL) and four heifers post-pubertal (i.e., POST; 2 consecutive progesterone values >1 ng/mL) [48], [57]. Day of puberty, for POST heifers was defined as the second consecutive day when serum progesterone was >1 ng/mL [47]. When the first heifer of the cohort of eight heifers obtained puberty, tissues from four randomly chosen pre-pubertal heifers were harvested. It then took approximately seven months for the remaining heifers to achieve puberty (i.e., POST). Tissues were harvested from PRE and POST heifers via slaughter in the New Mexico State University Meats Laboratory. Post-pubertal heifers were slaughtered on day 10 (i.e., mid-luteal phase) of the estrous cycle. Serum concentration of progesterone for the PRE and POST heifers were 0.5±0.3 and 7.1±1.0 ng/mL, respectively. Associated data collected during tissue harvest of PRE and POST heifers were body weights of 290 and 355±30 kg and days of age of 353 and 437±25, respectively.

### Tissue sampling

Heifers were stunned via captive bolt through the parietal bone of the head as to protect the integrity of the lower brain as described by Narro et al. [82]. From the lower brain, the hypothalamus (HYP) spanning from the optic chiasm to the arcuate nucleus was collected prior to collecting the anterior and posterior pituitary gland (PIT). Evisceration allowed collection of a 2 cm section of the lateral lobe of the liver (LIV) and the reproductive tract. Specifically, uterus (UTE) and endometrium (END) ipsilateral to the ovary (OVA) containing the *corpus luteum* was collected for use in this study from POST heifers. The region of the uterus and endometrium harvested spanned 6 to 8 cm from the ovarian pedicle. These same tissues were collected from the PRE heifers; however, they did not possess a *corpus luteum*, so tissues were collected from the side of the uterus with the larger ovary. A one cm section of the *longissimus dorsi* muscle (LDM) between the 12^th^ and 13^th^ rib and adipose tissue (FAT) was collected from the abdominal cavity adjacent to the spine and pelvis was also collected from both PRE and POST heifers. All tissues were collected within 15 min post mortem, snap-frozen in liquid nitrogen, and then stored at −80°C until processing. During tissue sampling and laboratory processing, we failed to collect or process three tissue samples (i.e., 2 END and 1 PIT). Therefore, a total of 61 tissue samples were available for RNA-Seq analyses (i.e., 8 HYP, 7 PIT, 8 LIV, 8 LDM, 8 FAT, 8 UTE, 6 END, and 8 OVA).

### RNA extraction and sequencing

Duplicate 10 g samples of each tissue were grounded in liquid nitrogen with mortar and pestle. From the ovary of POST heifers, this included tissue composing antral follicles and the corpus luteum, whereas from PRE heifers, this only included tissue containing antral follicles. Total RNA was purified using a Trizol protocol (Invitrogen, Carlsbad, CA). Quality was evaluated using the RNA Integrity Number (RIN) value from the Experion automated electrophoresis system (BioRad, Hercules, CA). The RIN values ranged from 7.6 to 9.8 in the tissues samples; lower RIN values were observed in adipose samples. As described by Cánovas et al. [83], mRNA was purified, fragmented, and converted to cDNA. Adapters were ligated to the ends of double-stranded cDNA and PCR amplified to create libraries. These procedures were executed with the TruSeq RNA Sample Preparation kit (Illumina, San Diego, CA).

Sequencing was completed with an Illumina HiSeq analyzer that yielded 100 bp single read sequences with exception of two hypothalamus samples done earlier on an Illumina GA II sequencer that yielded 36 bp sequence reads. Sequence reads were assembled to the annotated UMD3.1 bovine reference genome (release 74; ftp://ftp.ensembl.org/pub/release-74/genbank/bos_taurus/). Quality control and RNA-Seq expression analysis was performed using procedures described by Cánovas et al. [84] and using the CLC Genomics workbench software (CLC Bio, Aarhus, Denmark). Transcript levels were quantified in reads per kilobase of exon per million reads (RPKM). By normalizing for RNA length and total reads in each sample, the RPKM measure facilitated comparisons of transcript levels both within and between tissues [24]. In the present study, we used a threshold of RPKM ≥0.2 to select genes expressed in a given sample [22], [23].

### Normalization of RNA-Seq data

Oshlack et al. [85] acknowledged the optimality of generalized linear models as the logical extension to the modeling of RNA-Seq count data. In the present study, we first applied a base-2 log transformation of the RPKM reads. The log-transformation helps stabilizing the variance of RPKM values, an issue of critical importance as differential expression (DE) of particularly low counts can be easily biased without transformation [86]. We then adopted methodology initially proposed for the normalization of gene expression microarray intensity signals and based on mixed-model linear equations [87]. Accordingly, we fitted the following mixed effect model to the log-transformed RPKM values: **Y** *_ijtkp_*  =  µ + **L** *_i_* + **G** *_j_* + **GT** *_jt_* + **GA** *_jk_* + **GP** *_jp_* + **e** *_ijtkp_* where **Y** *_ijtkp_* represented the base-2 log-transformed RPKM value from the *i*-th library (with 61 levels), *j*-th gene (with 17,832 levels) in the *t*-th tissue (with 8 levels) of the *k*-th animal (with 8 levels) in the *p*-th physiological state (with 2 levels); µ was the overall mean, **L** *_i_* represented the fixed effect of the *i*-th library; **G** *_j_* represented the random effect of the *j*-th gene; **GT**, **GA**, and **GP** represented the random interaction effects of gene × tissue, gene × animal, and gene × physiological state, respectively; and **e** *_ijtkp_* was the residual term associated with the measurement in **Y** *_ijtkp_*. Using standard stochastic assumptions, the effects of **G**, **GT**, **GA**, **GP** and **e** were assumed to follow a normal distribution with zero mean and between-gene, between-gene within-tissue, between-gene within-animal, between-gene within-physiological state and within-gene components of variance, respectively. Restricted maximum likelihood estimates of variance components and solutions to model effects were obtained using VCE6 software (ftp://ftp.tzv.fal.de/pub/vce6/). The linear combination of solutions **G** + **GT** + **GA** + **GP** were used to obtain the normalized mean expression of each gene in each of the samples under scrutiny. In order to validate the optimality of the normalization approach, the normalized mean expression of the 17,832 genes across the 61 libraries was subjected to hierarchical cluster analysis using the PermutMatrix software [88]. Following normalization, we used a combination of parametric and non-parametric approaches for the identification of differentially expressed (DE) and tissue-specific (TS) genes, respectively.

### Identification of differentially expressed genes

For each gene in *i* (*i* = 1, …, 17,832) and based on the above-mentioned solutions to the gene × physiological state interactions (**GP**), we computed the following difference: 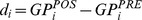



Large positive (or large negative) values of *d_i_* are likely to indicate that the *i*-th gene is up-regulated (or down-regulated) at puberty. Therefore, the vector with the difference (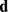) between the normalized mean expression of all genes in the two physiological states (PRE and POST) was computed as the measure of (possible) DE for each tissue. Using procedures of McLachlan et al. [89] in order to identify DE genes, we fitted a two-component normal mixture model as follows:
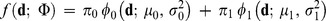
where 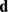 denoted the vector of DE measures for all the genes, and the two components in the mixtures correspond to: 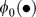 for the empirical null normal density with mean 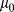 (not necessarily zero) and variance 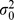 (not necessarily one), encapsulated the non-DE genes and 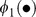 for the non-null distribution corresponding to DE genes. Finally, the mixing proportions 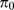 and 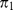 were constrained to be non-negative and sum to unity. Across the eight tissues, parameters of the mixture model were estimated using the EMMIX-GENE software [90] and an estimated experiment-wise false discovery rate (FDR) of <1% was used as the threshold for determining which genes were DE.

### Identification of tissue specific genes

We used the method of Schug et al. [91] to identify tissue specific (TS) genes based on Shannon entropy. Accordingly, the tissue specificity of gene *i* in tissue *t* was computed from the expression of gene *i* in tissue *t* relative to the expression of gene *i* in all tissues as follows:
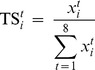
where 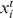 represented the expression of gene *i* in tissue *t* averaged across all libraries. In the denominator, the summation goes to 8 for as many tissues under scrutiny and of course the proportions in 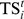 sum to unity. These proportions were interpreted as the probability of a given gene to belong to a given tissue. They were combined to compute the Shannon's entropy of a gene's expression as follows:
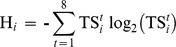




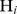
 ranged from 0 for genes expressed only in one tissue to 3 for genes equally expressed in all 8 tissues. The entropy values in 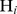 were not sensitive to absolute expression levels of genes in tissues because the values in 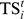 were relative expression levels. To circumvent this, a measure of categorical tissue specificity was computed as follows: 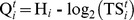.

The gene *i* was specific to tissue *t* as 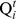 approaches zero according to this metric. However, on the other extreme, a gene equally expressed in all 8 tissues had a 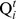  =  6 in all tissues. We used a permutation test, permuting the tissue labels in the original normalized data, to obtain the entropy that could be expected under random allocation of tissues. A total of 1,000 permutations were performed and the smallest entropy recorded as the threshold for tissue specificity at P<0.1%. Genes from the original data with an entropy value smaller than this threshold were considered TS genes. In order to validate the functional relationship of DE and TS genes, gene ontology (GO) enrichment analysis were performed using the procedures described in Fortes et al. [9], [10], [11] and the Gorilla software available at http://cbl-gorilla.cs.technion.ac.il/.

### Identification of key gene regulators

We employed two approaches for the identification of key gene regulators. For the first approach, we used regulatory impact factor (RIF) metrics described by Reverter et al. [92] to identify the regulators with the highest evidence of contributing to DE and (or) TS in the two physiological states, PRE and POST. We mined the animal transcription factor (TF) database of Zhang et al. [93], freely available at http://www.bioguo.org/AnimalTFDB/. From this database, we downloaded 1,329 TFs in 69 families, 99 chromatin remodeling factors and 255 transcription co-factors of *Bos taurus*.

Using the RIF metrics, these regulators were contrasted against a unique list of genes that were either DE or TS. While the original implementation of the RIF metrics involved the comparison of the TF with the DE genes, the exact same algebra was adapted to the comparison of the TF with the TS genes (or any other group of genes for that matter) as long as an experimental contrast was defined. Herein, the experimental contrast was PRE versus POST and the RIF metrics for the *r*-th regulator (*r* = 1, 2, …, 1329) were computed using the following formulae:
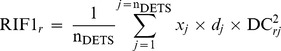
and
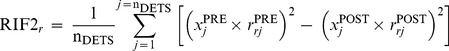
where n_DETS_ represented the number of genes that were either DE or TS; 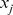 was the average expression of the *j*-th DE or TS gene across all samples; 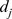 was the DE of the *j*-th gene in the PRE- *vs.* POST-puberty contrast; 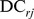 was the differential co-expression between the *r*-th regulator and the *j*-th DE or TS gene, and computed from the difference between 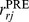 and 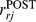, the correlation co-expression between the *r*-th regulator and the *j*-th DE or TS gene in the PRE- and POST-puberty samples, respectively; finally, 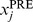 and 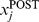 represented the average expression of the j-th DE or TS gene in the PRE- and POST-puberty samples, respectively;

In the second approach to identify key regulators, we used the methodology described in Gu et al. [13] based on promoter sequence analysis of the DE and (or) TS genes to search for motifs corresponding to transcription factor binding sites (TFBS) of known TF. Specifically, a total of 60,131 promoter sequences derived from 22,050 genes were downloaded from the bovine genome-wide promoter sequence database of Genomatix (http://www.genomatix.de/; ElDorado Btau 4, v-07-09). To ensure only high confidence promoters were selected, we applied the concept of orthologous promoters [94] and retained only those promoters for which phylogenetically conserved sequences were documented in both the human and mouse genomes. This resulted in the identification of 39,696 promoter sequences distributed over 13,623 genes. We subsequently applied a threshold of 1 (100% confidence) to core and matrix similarities [95] to identify a final set of 310,316 high confidence TFBS that were used for integration with the RNA-Seq data.

### Identification of key genes harboring SNP

The PRE and POST heifers used in this study were derived from a population of Brangus heifers that were included in GWAS of heifer fertility traits [11]. These studies tested associations between the SNP on the Illumina BovineSNP50 BeadChip (54,001 ver. 1; Illumina, San Diego, CA) and the traits of FSC and HPG. Heifers that conceived early in a breeding season, were considered as early puberty within their first (i.e., yearling) breeding season. The SNP identified in previous studies involving ACL were also included in this effort [11], [39] and cumulatively results of these three publications were also used to identify target genes. Specifically, the 1,555 SNP reported by Fortes et al (11) predicting FSC and the 169 SNP from study of Brahman cattle and 84 SNP from study of Australian tropical composite cattle described by Hawken et al (39) were used in these GWAS efforts. We also combined results from GWAS (p<1%; <10 kb) with the 17,832 genes expressed in at least one tissue and physiological state from RNA-Seq data.

### Gene networks and functional analysis

Gene networks analysis was performed using the partial correlation and information theory (PCIT) algorithms and software described by Reverter and Chan [96]. This is a soft-thresholding methodology that exploits the twin concepts of PCIT. In brief, it explores relationships between all possible triplets of genes as to determine informative correlations between gene pairs once the numerical influence of other genes in the system were estimated. The networks were then viewed with Cytoscape [97] revealing the highly connected genes and hubs (i.e., 2 SD as a nominal threshold, *P*<0.01) [98]. Also, for DE and TS genes, GO enrichment analysis was performed using GOrilla software available at http://cbl-gorilla.cs.technion.ac.il/ as to gain understanding of the function of the nodes in the networks.

From within the larger networks, comprising DE genes, TS genes, TF and genes harboring SNP related to fertility, we explored a series of subnetworks deemed to be of particular relevance as defined by the connectivity degree and identity of their gene members. In particular, two of these were: (1) the subnetwork comprised by the best trio of TF in terms of their ability to expand the majority of the topology of the entire networks, and (2) the subnetwork comprised by the significant coexpression correlations between a highly ranked TF and a highly DE gene in either the hypothalamus or the pituitary gland.

## Supporting Information

Table S1 The 2,450 genes and their average expression level (log^2^) involved in network analyses. These genes were differentially expressed (DE), tissue specific (TS), a transcription factor (TF) and (or) contained a SNP detected with GWAS in PRE and POST heifers.
(XLSX)

Table S2 Genes captured in the cattle network concordant with the list of 30 new loci for age at menarche in humans [79].
(DOCX)

## References

[1] Dorn LD, Biro FM (2011) Puberty and its measurement: a decade in review. J Res Adolscence 21: 180–195.

[2] Burns BM, Fordyce G, Holroyd RG (2010) A review of factors that impact on the capacity of beef cattle females to conceive, maintain a pregnancy and wean a calf-Implications for reproductive efficiency in northern Australia. Anim Reprod Sci 122: 1–22. 20447780 10.1016/j.anireprosci.2010.04.010 PMC7131258

[3] Day ML, Nogueira GP (2013) Management of age at puberty in beef heifers to optimize efficiency of beef production. Anim Front 3: 6–11.

[4] Rodrigues HD, Kinder JE, Fitzpatrick LA (2002) Estradiol regulation of luteinizing hormone secretion in heifers of two breed types that reach puberty at different ages. Biol Reprod 66: 603–609. 11870064 10.1095/biolreprod66.3.603

[5] Nogueira GP (2004) Puberty in South American Bos indicus (Zebu) cattle. Anim Reprod Sci 82–83: 361–372. 10.1016/j.anireprosci.2004.04.007 15271466

[6] Elsik CG, Tellam RL, Worley KC (2009) The genome sequence of tuarine cattle: a window to ruminant biology and evolution. Science 324: 522–528. 19390049 10.1126/science.1169588 PMC2943200

[7] Reverter A, Fortes MRS (2013) Breeding and Genetics Symposium: building single nucleotide polymorphism-derived gene regulatory networks: toward functional genomewide association studies. J Anim Sci 91: 530–536. 23097399 10.2527/jas.2012-5780

[8] Snelling WM, Cushman RA, Keele JW, C Maltecca, Thomas MG, et al (2013) Breeding and Genetics Symposium: networks and pathways to guide genomic selection. J Anim Sci 91: 537–552. 23097404 10.2527/jas.2012-5784

[9] Fortes MRS, Reverter A, Zhang Y, Collis E, Nagaraj SH, et al (2010) Association weight matrix for the genetic dissection of puberty in beef cattle. Proc Natl Acad Sci 107: 13642–13647. 20643938 10.1073/pnas.1002044107 PMC2922254

[10] Fortes MRS, Reverter A, Nagaraj SH, Zhang Y, Jonsson NN, et al (2011) A single nucleotide polymorphism-derived regulatory gene network underlying puberty in 2 tropical breeds of beef cattle. J Anim Sci 89: 1669–1683. 21357453 10.2527/jas.2010-3681

[11] Fortes MRS, Snelling WM, Reverter A, Nagaraj SH, Lehnert SA, et al (2012a) Gene network analyses of first service conception in Brangus heifers: use of genome and trait associations, hypothalamic-transcriptome information, and transcription factors. J Anim Sci 90: 2894–2906. 22739780 10.2527/jas.2011-4601

[12] Hudson NJ, Reverter A, Wang Y, Greenwood PL, Dalrymple BP (2009) Inferring the transcriptional landscape of bovine skeletal muscle by integrating co-expression networks. PloS One 4: e7249. 19794913 10.1371/journal.pone.0007249 PMC2749936

[13] Gu Q, Nagaraj SH, Hudson NJ, Dalrymple BP, Reverter A (2011) Genome-wide patterns of promoter sharing and co-expression in bovine skeletal muscle. BMC Genomics 12: 23. 21226902 10.1186/1471-2164-12-23 PMC3025955

[14] Sun W, Hudson WJ, Reverter A, Waardenberg AJ, Tellam RL, et al (2012) An always correlated gene expression landscape for ovine skeletal muscle, learnt from comparison with “equivalent” bovine landscape. BMC Res Notes 5: 632. 23148653 10.1186/1756-0500-5-632 PMC3543716

[15] Vidal M, Cusick ME, Barabasi AL (2011) Interactome networks and human disease. Cell 144: 986–998. 21414488 10.1016/j.cell.2011.02.016 PMC3102045

[16] Barabasi AL, Gulbahce N, Loscalzo J (2011) Network medicine: a network-based approach to human disease. Nature Rev Genet 12: 56–68. 21164525 10.1038/nrg2918 PMC3140052

[17] Chan SY, Loscalzo J (2012) The emerging paradigm of network medicine in the study of human disease. Circul Res J Am Heart Assoc 111: 359–374. 10.1161/CIRCRESAHA.111.258541 PMC3425394 22821909

[18] Peters SO, Kizilkaya K, Garrick DJ, Fernando RL, Reecy JM, et al (2012) Bayesian quantitative loci inference from whole genome analyses of growth and yearling ultrasound measures of carcass traits in Brangus heifers. J Anim Sci 90: 3398–3409. 23038745 10.2527/jas.2012-4507

[19] Peters SO, Kizilkaya K, Garrick DJ, Fernando RL, Reecy J, et al (2013) Heritability and Bayesian genome-wide association study of first service conception and pregnancy in Brangus heifers. J Anim Sci 91: 605–612. 23148252 10.2527/jas.2012-5580

[20] Rapaport F, Khanin R, Lian Y, Pirun Y, et al (2013) Comprehensive evaluation of differential gene expression analysis methods for RNA-Seq data. Genome Biol 14: R95. 24020486 10.1186/gb-2013-14-9-r95 PMC4054597

[21] Medrano JF, Rincon G, Islas-Trejo A (2010) Comparative analysis of bovine milk and mammary gland transcriptome using RNA-Seq. Proc 9^th^ World Cong Appl Livestock Prod, Lepizig, Germany, #852, http://www.kongressband.de/wcgalp2010/assets/pdf/0852.pdf.

[22] Wickramasinghe S, Hua S, Rincon G, Ilas-Trejo A, German JB, et al (2011) Transcriptome profiling of bovine milk oligosaccharide metabolism genes using RNA-sequencing. PLoS One 6: e18895. 21541029 10.1371/journal.pone.0018895 PMC3081824

[23] Wickramasinghe S, Rincon G, Islas-Trejo A, Medrano JF (2012) Transcriptional profiling of bovine milk using RNA sequencing. BMC Genomics 13: 45. 22276848 10.1186/1471-2164-13-45 PMC3285075

[24] Mortazavi A, Williams BA, McCue K, Schaeffer L, Wold B (2008) Mapping and quantifying mammalian transcriptomes by RNA-Seq. Nat Methods 5: 621–628. 18516045 10.1038/nmeth.1226 PMC13303166

[25] Zieba DA, Amstalden M, Williams GL (2005) Regulatory roles of leptin in reproduction and metabolism: a comparative review. Dom Anim Endocrinol 29: 166–185. 10.1016/j.domaniend.2005.02.019 15927772

[26] Amstalden M, Alves BRC, Liu S, Cardoso RC, Williams GL (2011) Neuroendocrine pathways mediating nutritional acceleration of puberty: insights from ruminant models. Front Endocrinol 2: 109 doi:10.3389/fendo.2011.00109 10.3389/fendo.2011.00109 PMC3356117 22654842

[27] Dobson H, Smith RF (2000) What is stress, and how does it affect reproduction? Anim Reprod Sci. 60–61: 743–52. 10.1016/s0378-4320(00)00080-4 10844239

[28] Gimpl G, Fahrenholz F (2001) The oxytocin receptor system: structure, function, and regulation. Physiol Rev 81: 629–683. 11274341 10.1152/physrev.2001.81.2.629

[29] Gabbay-Benziv R, Ao A, Fisch B, Zhang L, Oron G, et al (2012) Vasoactive intestinal peptide and its receptors in human ovarian cortical follicles. PLoS One 7: e37015. 22623971 10.1371/journal.pone.0037015 PMC3356394

[30] Chen N, Li Y, Wang W, Ma Y, Yang D, et al (2013) Vasoactive intestinal peptide can promote the development of neonatal rat primordial follicles during in vitro culture. Biol Reprod 88: 12. 23175772 10.1095/biolreprod.111.098335

[31] Bafunno V, Santacroce R, Margaglione M (2011) The risk of occurrence of venous thrombosis: focus on protein Z. Thromb Res 128: 508–515. 21885093 10.1016/j.thromres.2011.08.007

[32] Almawi WY, Al-Shaikh FS, Melemedjian OK, Almawi AW (2013) Protein Z, an anticoagulant protein with expanding role in reproductive biology. Reproduction 146: R73–80. 23690629 10.1530/REP-13-0072

[33] Uechi T, Tanaka T, Kenmochi N (2001) A complete map of the human ribosomal protein genes: assignment of 80 genes to the cytogenetic map and implications for human disorders. Genomics 72: 223–230. 11401437 10.1006/geno.2000.6470

[34] Sugihara Y, Sadohara E, Yonezawa, Kugo M, Oshima T, et al (2013) Identification and expression of an autosomal paralogue of ribosomal protein S4, X-linked, in mice: potential involvement of testis-specific ribosomal proteins in translation and spermatogensis. Gene 521: 91–99. 23500592 10.1016/j.gene.2013.02.040

[35] Petraglia F, Imperatore A, Challis JRG (2010) Neuroendocrine mechanisms in pregnancy and parturition. Endo Rev 31: 783–816. 10.1210/er.2009-0019 20631004

[36] Bathgate RAD, Halls ML, Van der Westhuizen ET, Callander GE, Kocan M, Summers RJ (2012) Relaxin family peptides and their receptors. Physiol Rev 93: 405–480. 10.1152/physrev.00001.2012 23303914

[37] Vuong C, Van Uum SHM, O'Dell LE, Lutfy K, Friedman TC (2010) The effects of opioids and opioid analogs and human endocrine systems. Endo Rev 31: 98–132. 10.1210/er.2009-0009 PMC2852206 19903933

[38] Subiran N, Casis L, Irazusta J (2011) Regulation of male fertility by the opioid system. Mol Med 17: 846–853. 21431247 10.2119/molmed.2010.00268 PMC3146603

[39] Hawken RJ, Zhang YD, Fortes MRS, Collis E, Barris WC, Corbet NJ, et al (2012) Genome-wide association studies of female reproduction in tropically adapted beef cattle. J Anim Sci 90: 2894–2906. 22100599 10.2527/jas.2011-4410

[40] Fortes MRS, Kemper K, Sasazaki S, Reverter A, et al (2013) Evidence for pleiotropism and recent selection in the PLAG1 region in Australian Beef cattle. Anim Geneti 44: 636–647. 10.1111/age.12075 23909810

[41] Larder R, Clark DD, Miller NL, Mellon PL (2011) Hypothalamic dysregulation and infertility in mice lacking the homeodomain protein Six6. J Neurosci 31: 426–438. 21228153 10.1523/JNEUROSCI.1688-10.2011 PMC3103738

[42] Tan O, Carr BR, Beshay VE, Bukulmez O (2013) The extrapituitary effects of GnRH antagonists and their potential clinical implications: a narrated review. Reprod Sci 20: 16–25. 23012318 10.1177/1933719112459244

[43] D'Elia A, Tell G, Paron I, Pellizzari L, Lonigro, Damante G (2001) Missense mutations of homeoboxes: a review. Hum Mut 18: 361–374. 11668629 10.1002/humu.1207

[44] Raetzman LT, Ward R, Camper SA (2002) Lhx4 and Prop1 are required for cell survival and expansion of the pituitary primordia. Development 129: 4229–4239. 12183375 10.1242/dev.129.18.4229

[45] Scully KM, Rosenfeld MG (2002) Pituitary development: regulatory codes in mammalian organogensis. Science 295: 2231–2235. 11910101 10.1126/science.1062736

[46] Kogata N, Zvelebil M, Howard BA (2013) Neuregulin 3 and erbb signalling networks in embryonic mammary gland development. J Mammary Gland Biol Neoplasia 18: 149–54. 23649700 10.1007/s10911-013-9286-4

[47] Chazenbalk G, Chen YH, Heneidi S, Lee JM, Pall M, et al (2012) Abnormal expression of genes involved in inflammation, lipid metabolism, and Wnt signaling in the adipose tissue of polycystic ovary syndrome. J Clin Endocrinol Metab 97: E765–770. 22344199 10.1210/jc.2011-2377 PMC3339894

[48] Shirley KL, Thomas MG, Keisler DH, Hallford DM, Montrose DM, et al (2006) Case study: a Chihuahuan Desert Brangus breeding program: feed efficiency, metabolic hormones, and puberty in heifers sired by bulls with different expected progeny differences for growth and scrotal circumference. Prof Anim Sci 22: 48–58.

[49] Garrett AJ, Rincon G, Medrano JF, Elzo MA, Silver GA, et al (2008) Promoter region of the bovine growth hormone receptor gene: Single nucleotide polymorphism discovery in cattle and association with performance in Brangus bulls. J Anim Sci 86: 3315–3323. 18676722 10.2527/jas.2008-0990

[50] Luna-Nevarez P, Rincon G, Medrano JF, Riley DG, Chase CC, et al (2011) Single nucleotide polymorphisms in the growth hormone – insulin like growth factor axis in straightbred and crossbred Angus, Brahman, and Romosinuano heifers: population genetic analyses and association of genotypes with reproductive phenotypes. J Anim Sci 89: 926–934. 21183713 10.2527/jas.2010-3483

[51] O'Flynn O'Brien KL, Varghese AC, Agarwal A (2010) The genetic causes of male factor infertility: a review. Fert Steril 93: 1–12. 10.1016/j.fertnstert.2009.10.045 20103481

[52] Satchell L, Glister C, Bleach EC, Glencross RG, Bicknell AB, et al (2013) Ovarian expression of insulin-like peptide 3 (INSL3) and its receptor (RXFP2) during development of bovine antral follicles and corpora lutea and measurement of circulating INSL3 levels during synchronized estrous cycles. Endocrinology 154: 1897–1906. 23546605 10.1210/en.2012-2232

[53] Cánovas A, Quintanilla R, Amills M, Pena RN (2010b) Muscle transcriptomic profiles in pigs with divergent phenotypes for fatness traits. BMC Genomics 11: 372. 20540717 10.1186/1471-2164-11-372 PMC2894043

[54] Narukami T, Sasazaki S, Oyama K, Nogi T, Taniguchi M, et al (2011) Effect of DNA polymorphisms related to fatty acid composition in adipose tissue of Holstein cattle. Anim Sci J 82: 406–411. 21615833 10.1111/j.1740-0929.2010.00855.x

[55] Lee SH, van der Werf JH, Park EW, Oh SJ, Gibson JP, et al (2010) Genetic polymorphisms of the bovine fatty acid binding protein 4 gene are significantly associated with marbling and carcass weight in Hanwoo (Korean Cattle). Anim Genet 41: 442–444. 20331595 10.1111/j.1365-2052.2010.02024.x

[56] Tabandeh MR, Hosseini A, Saeb M, Kafi M, Saeb S (2010) Changes in the gene expression of adiponectin and adiponectin receptors (AdipoR1 and AdipoR2) in ovarian follicular cells of dairy cow at different stages of development. Theriogenology 73: 659–669. 20047754 10.1016/j.theriogenology.2009.11.006

[57] Lopez R, Thomas MG, Hallford DM, Keisler DH, Silver GA, et al (2006) Case study: metabolic hormone profiles and evaluation of associations of metabolic hormones with body fat and reproductive characteristics of Angus, Brangus, and Brahman heifers. Prof Anim Sci 22: 273–282.

[58] Luna-Nevarez P, Bailey DW, Bailey CC, VanLeeuwen DM, Enns RM, et al (2010) Growth characteristics, reproductive performance, and evaluation of their associative relationships in Brangus cattle managed in a Chihuahuan Desert production system. J Anim Sci 88: 1891–1904. 20154157 10.2527/jas.2009-2541

[59] Ulbrich SE, Frohlich T, Schulke K, Englberger E, Waldschmitt N, et al (2009) Evidence for estrogen-dependent uterine serpin (SERPINA14) expression during estrus in the bovine endometrial glandular epithelium and lumen. Biol Reprod 81: 795–805. 19494250 10.1095/biolreprod.108.075184

[60] Martinez-Giner M, Noguera JL, Balcells I, Alves E, Varona L, et al (2011) Expression study on the porcine PTHLH gene and its relationship with sow teat number. J Anim Breed Genet 128: 344–353. 21906180 10.1111/j.1439-0388.2011.00925.x

[61] Torricelli M, Voltolini C, Novembri R, Bocchi C, Di Tommaso M, et al (2012) Activin A and its regulatory molecules in placenta and fetal membranes of women with preterm premature rupture of the membranes associated with acute chorioamnionitis. Am J Reprod Immunol 68: 392–399. 22845186 10.1111/j.1600-0897.2012.01180.x

[62] Gregory SJ, Kaiser UB (2004) Regulation of gonadotropins by inhibin and activin. Semin Reprod Med 22: 253–267. 15319828 10.1055/s-2004-831901

[63] Matorras R, Osuna C, Exposito A, Crisol L, Pijoan JI (2011) Recombinant FSH versus high purified FSH in intrauterine insemination: systematic review and metaanalysis. Fert Steril 95: 1937–1942.e3. 10.1016/j.fertnstert.2011.02.030 21429486

[64] Roth CL, Mastronardi C, Lomniczi A, Wright H, Cabrera R, et al (2007) Expression of a tumor-related gene network increases in the mammalian hypothalamus at the time of female puberty. Endocrinology 148: 5147–5161. 17615149 10.1210/en.2007-0634

[65] Ojeda SR, Dubay C, Lomniczi A, Kaidar G, Matagne V, et al (2010) Gene networks and the neuroendocrine regulation of puberty. Mol Cell Endocrinol 324: 3–11. 20005919 10.1016/j.mce.2009.12.003 PMC2888991

[66] Lomniczi A, Wright H, Castellano JM, Sonmez K, Ojeda SR (2013) A system biology approach to identify regulatory pathways underlying the neuroendocrine control of female puberty in rats and non-human primates. Horm Behav 64: 175–86. 23998662 10.1016/j.yhbeh.2012.09.013 PMC3933372

[67] Bione S, Rizzolio F, Sala C, Ricotti R, Goegan M, et al (2004) Mutation analysis of two candidate genes for premature ovarian failure, DACH2 and POF1B. Hum Reprod 19: 2759–2766. 15459172 10.1093/humrep/deh502

[68] Lam EWF, Brosens JJ, Gomes AR, Koo CY (2013) Forkhead box proteins: tuning forks for transcriptional harmony. Nat Rev Cancer 13: 482–495. 23792361 10.1038/nrc3539

[69] Thackray VG (2014) Fox tales: regulation of gonadotropin gene expression by forkhead transcription factors. Mol Cell Endocrinol 385: 62–70. 24099863 10.1016/j.mce.2013.09.034 PMC3947687

[70] Suzumori N, Pangas SA, Rajkovic A (2007) Candidate genes for premature ovarian failure. Curr Med Chem 14: 353–357. 17305537 10.2174/092986707779941087

[71] Bin G, Jiarong Z, Shihao W, Xiuli S, Cheng X, et al (2012) Aire promotes the self renewal of embryonic stem cells through Lin28. Stem Cell Develop 21: 2878–2890. 10.1089/scd.2012.0097 PMC3464070 22540148

[72] Connell MT, Owen CM, Segars JH (2013) Genetic syndromes and genes involved in the development of the female reproductive tract: a possible role for gene therapy. J Genet Syndr Gene Ther 4: 1000127. 10.4172/2157-7412.1000127 PMC4264624 25506511

[73] Shah N, Sukumar S (2010) The Hox genes and their roles in oncogenesis. Nat. Rev. Cancer 10: 361–371. 10.1038/nrc2826 20357775

[74] Markholt S, Grondahl ML, Ernst EH, Andersen CY, Ernst E, Lykke-Hartmann K (2012) Global gene analysis of oocystes from early stages in human folliculogenesis show high expression of novel genes in reproduction. Mol Human Reprod 18: 96–110. 10.1093/molehr/gar083 22238370

[75] Dupre A, Haccard O, Jessus C (2011) Review article: MOS in the oocyte: how to use MAPK independently of growth factors and transcription to control meiotic divisions. J Signal Trans 2011: 350412. 10.1155/2011/350412 PMC3101788 21637374

[76] Rogers RL, Van Seuningen I, Gould J, Hertzog PJ, Naylor MJ, Pritchard MA (2010) Transcript profiling of Elf5+/− mammary glands during pregnancy identifies novel targets of ELF5. PLoS One 5: e13150. 20949099 10.1371/journal.pone.0013150 PMC2951341

[77] Ounzain S, Bowen S, Patel C, Fujita R, Heads RJ, et al (2011) Proliferation-associated POU4F2/Brn-3b transcription factor expression is regulated by oestrogen through ERα and growth factors via MAPK pathway Breast Cancer Res. 13: R5. 10.1186/bcr2809 PMC3109571 21241485

[78] Tang K-Q, Li S-J, Yang W-C, Yu J-N, et al (2011) An MspI polymorphism in the inhibin alpha gene and its association with superovulation traits in Chinese Holstein cows. Mol Biol Rep 38: 17–21. 20238172 10.1007/s11033-010-0072-8

[79] Moreno-Sanchez N, Rueda J, Reverter A, Carabano MJ, Diaz C (2012) Muscle-specific gene expression is underscored by differential stressor responses and coexpression changes. Funct Integr Genomics 12: 93–103. 21881922 10.1007/s10142-011-0249-9

[80] Elks CE, Perry JRB, Sulem P, Chasman, et al (2010) Thirty new loci for age at menarche identified by a meta-analysis of genome-wide association studies. Nat Genet 42: 1077–1085. 21102462 10.1038/ng.714 PMC3140055

[81] Schneider FA, Hallford DM (1996) Use of a rapid progesterone radio-immunoassay to predict pregnancy and fetal numbers in ewes. Sheep Goat Res J 12: 33–38.

[82] Narro LA, Thomas MG, Silver GA, Rozeboom KJ, Keisler DH (2003) Body composition, leptin, and the leptin receptor and their relationship to the growth hormone (GH) axis in growing wethers treated with zeranol. Domest Anim Endocrinol 24: 243–255. 12642164 10.1016/s0739-7240(02)00239-4

[83] Cánovas A, Rincon G, Islas-Trejo A, Wickramasinghe S, Medrano JF (2010a) SNP discovery in the bovine milk transcriptome using RNA-Seq technology. Mamm Genome 21: 592–598. 21057797 10.1007/s00335-010-9297-z PMC3002166

[84] Cánovas A, Rincon G, Islas-Trejo A, Jimenez-Flores R, Laubscher A, et al (2013) RNA sequencing to study gene expression and single nucleotide polymorphism variation associated with citrate content in cow milk. J Dairy Sci 96: 2637–2648. 23403202 10.3168/jds.2012-6213

[85] Oshlack A, Robinson MD, Young MD (2010) From RNA-seq reads to differential expression results. Genome Biol 11: 220. 21176179 10.1186/gb-2010-11-12-220 PMC3046478

[86] Bullard JH, Purdom E, Hansen KD, Dudoit S (2010) Evaluation of statistical methods for normalization and differential expression in mRNA-Seq experiments. BMC Bioinformatics 11: 94. 20167110 10.1186/1471-2105-11-94 PMC2838869

[87] Reverter A, Barris W, McWilliam S, Byrne KA, Wang YH, et al (2005) Validation of alternative methods of data normalization in gene co-expression studies. Bioinformatics 21: 1112–1120. 15564293 10.1093/bioinformatics/bti124

[88] Caraux G, Pinloche S (2005) PermutMatrix: a graphical environment to arrange gene expression profiles in optimal linear order. Bioinformatics 21: 1280–1281. 15546938 10.1093/bioinformatics/bti141

[89] McLachlan GJ, Bean RW, Jones LB (2006) A simple implementation of a normal mixture approach to differential gene expression in multiclass microarrays. Bioinformatics 22: 1608–1615. 16632494 10.1093/bioinformatics/btl148

[90] McLachlan GJ, Bean RW, Peel D (2002) A mixture model-based approach to the clustering of microarray expression data. Bioinformatics 18: 413–422. 11934740 10.1093/bioinformatics/18.3.413

[91] Schug J, Schuller WP, Kappen C, Salbaum JM, Bucan M, et al (2005) Promoter features related to tissue specificity as measured by Shannon entropy. Genome Biol 6: R33. 15833120 10.1186/gb-2005-6-4-r33 PMC1088961

[92] Reverter A, Hudson NJ, Nagaraj SH, Perez-Enciso M, Dalrymple BP (2010) Regulatory impact factors: unraveling the transcriptional regulation of complex traits from expression data. Bioinformatics 26: 896–904. 20144946 10.1093/bioinformatics/btq051

[93] Zhang HM, Chen H, Liu W, Liu H, Gong J, et al (2012) AnimalTFDB: a comprehensive animal transcription factor database. Nucleic Acids Res 40: D144–149. 22080564 10.1093/nar/gkr965 PMC3245155

[94] Buske FA, Boden M, Bauer DC, Bailey TL (2010) Assigning roles to DNA regulatory motifs using comparative genomics. Bioinformatics 26: 860–866. 20147307 10.1093/bioinformatics/btq049 PMC2844991

[95] Cartharius K, Frech K, Grote K, Klocke B, Haltmeier M, et al (2005) MatInspector and beyond: promoter analysis based on transcription factor binding sites. Bioinformatics 21: 2933–2942. 15860560 10.1093/bioinformatics/bti473

[96] Reverter A, Chan EK (2008) Combining partial correlation and an information theory approach to the reversed engineering of gene co-expression networks. Bioinformatics 24: 2491–2497. 18784117 10.1093/bioinformatics/btn482

[97] Shannon P, Markiel A, Ozier O, Baliga NS, Wang, et al (2003) Cytoscape: A software environment for integrated models of biomolecular interaction networks. Genome Res 13: 2498–2504. 14597658 10.1101/gr.1239303 PMC403769

[98] Tyler AL, Asselbergs FW, Williams SM, Moore JH (2009) Shadow of complexity: what biological networks reveal about epistasis and pleiotropy. BioEssays 31: 220–227. 19204994 10.1002/bies.200800022 PMC3159922

