# Supplementary material for: Multi-Tissue Omics Analyses Reveal Molecular Regulatory Networks for Puberty in Composite Beef Cattle
Source: PLoS One. 2014 Jul 21;9(7):e102551. doi: 10.1371/journal.pone.0102551 (PMC4105537; doi:10.1371/journal.pone.0102551)
Supplement: Table S2 — Genes captured in the cattle network concordant with the list of 30 new loci for age at menarche in humans [79]. (DOCX) [file pone.0102551.s002.docx]

**Table S2.** Genes captured in the cattle network concordant with the list of 30 new loci for age at menarche in humans (Elks et al. 2010. Nat Genet. 42:1077-1085).

| **Gene** | ***Attributes** | | | | **Tissue** | **Ten most co-expressed genes** |
| --- | --- | --- | --- | --- | --- | --- |
|  | **DE** | **TS** | **TF** | **SNP** |  |  |
| BSX | 1 | 0 | 1 | 0 | HYP | CHRNA4,DLX1,GPR17,GRM5,KLK6,KRT81,KRT86,OLIG3,ONECUT3,bta-mir-935 |
| CRTC1 | 0 | 0 | 1 | 0 | HYP | ACE2,CAMK2B,COL18A1,CPN2,DIRAS1,GLP2R,NKIRAS1,STAC2,TLE4,XPNPEP2 |
| INHBA | 0 | 1 | 0 | 0 | OVA | AURKA,CST15,FAM54A,FDXR,HSD3B,KCNK18,KRT2,KRT73,MOS,STAR |
| NFAT5 | 0 | 0 | 1 | 0 | PIT | ATF2,C9H6orf165,CREB1,EFCAB12,EPS8L1,FAM227A,FSIP1,STK33,TEKT1,TOX |
| PCSK2 | 1 | 0 | 0 | 0 | HYP | AMZ1,ANKRD55,CXXC4,EGR4,GABRA2,HS6ST3,MMEL1,OTOS,RDH12,SCN8A |
| TMEM108 | 0 | 0 | 0 | 1 | PIT | GABRB3,GRID2IP,KIF3A,LGI1,LOC527068,MYT1L,SCN8A,SEZ6,SYNPR,ZNF239 |

*Attributes: (1) differentially expressed (DE) among pre- versus post-puberty; (2) tissue specific (TS); (3) transcription factors (TF); (4) genes harboring SNP associated with indicator traits of puberty (first service conception (FSC), heifer pregnancy (HPG), and age at puberty as measured by the presence of the first corpus luteum (ACL)). HYP (hypothalamus); PIT (pituitary gland); OVA (ovary).
